# Automated Physical Activity Support for Adults and Youth From Low-Income Communities: Single-Arm Pilot Study

**DOI:** 10.2196/76991

**Published:** 2026-06-05

**Authors:** Jordan A Carlson, Frank Materia, Mallory Moon, Suryeon Ryu, Cory Yeager, Chelsea Steel, Kacee Shields, Harpreet Singh Gill, Jannette Berkley-Patton, Delwyn Catley

**Affiliations:** 1 Center for Children's Healthy Lifestyles & Nutrition Children's Mercy Hospital Kansas City, MO United States; 2 Department of Otolaryngology-Head and Neck Surgery University of Kansas Medical Center Kansas City, KS United States; 3 Department of Population Health University of Kansas Medical Center Kansas City, KS United States; 4 Research Informatics Children's Mercy Hospital Kansas City, MO United States; 5 Department of Biomedical and Health Informatics School of Medicine University of Missouri–Kansas City Kansas City, MO United States; 6 Department of Psychology San Diego State University San Diego, CA United States

**Keywords:** African American, community engagement, medically underserved, text messaging, wearables

## Abstract

**Background:**

Mobile health (mHealth) interventions are growing in popularity, but less research has focused on low-income families, particularly interventions integrating wearable devices with automated personalized messages.

**Objective:**

We tested a preliminary wearable-integrated mHealth intervention with initial personalization elements among adults and youth from low-income urban communities, focusing on feasibility, acceptability, and preliminary evidence of physical activity behavior.

**Methods:**

Participants were 83 adults and 31 youth recruited through community health events held in low-income urban communities. Using a single-arm pre-post design, participants were enrolled into a 7-week beta-version mHealth intervention that integrated a Garmin activity monitor with automated text messages. Messages were sent 4 days/week and focused on increasing step counts using theory-based behavior change techniques related to goal setting, self-monitoring, reinforcement, contextual factors, and self-efficacy. Most messages were personalized by including calculations based on the step-count and step-goal data, using branching logic, and using 2-way question-and-response messages. Feasibility measures included enrollment, retention, fidelity of message delivery, and adherence to wearing the Garmin device. Acceptability measures included survey items and engagement with responding to 2-way messages. Changes in daily steps were explored using mixed-effects linear regression.

**Results:**

Enrollment and eligibility rates were 64% (84/132, adults) and 63% (31/49, youth), retention for physical activity measures was 84% (70/83) and 77% (24/31), and 99% (3910/3955) of the intended messages were delivered. Adults and youth adhered to wearing the Garmin on 82% (45/56) and 79% (44/56) of the study days, respectively. Overall acceptability ratings were 83% to 100%, with 97% (75/77) of adults and 100% (27/27) of youth indicating they would recommend the program. Adults and youth replied to a mean of 2.6 (SD 2.2) and 3.2 (SD 2.7) of the 7 text messages that asked for a reply, with higher engagement among adults who participated with their child. Pre-post changes in daily steps were β=240 (95% CI –387 to 866) for adults and β=413 (95% CI –877 to 1703) among youth, with larger changes observed among those in the highest tertile of engagement (adults: β=584, 95% CI –784 to 1952; n=19; youth: β=941, 95% CI –827 to 2709; n=11) and those who were meeting less than two-thirds of the physical activity guideline at baseline (adults: β=609, 95% CI –30 to 1247; n=47; youth: β=1406, 95% CI –94 to 2907; n=22).

**Conclusions:**

Personalized mHealth physical activity interventions integrating wearable step trackers with automated text messaging appear to be feasible and acceptable among adults and youth from low-income communities. Step-count findings show promise for the intervention’s ability to support individuals who are further from meeting physical activity guidelines and warrant more research among parent–child dyads. Overall, findings support additional research to optimize and evaluate similar interventions within this population group using fully powered randomized controlled trials.

**Trial Registration:**

ClinicalTrials.gov NCT05110508; https://clinicaltrials.gov/ct2/show/NCT05110508

## Introduction

Increasing physical activity is central to the prevention and control of many diseases and promotion of general wellness for adults and youth [1]. Physical activity interventions are especially important for low-income population groups, which can be underresourced and have been shown to engage in less leisure-time physical activity and experience higher rates of related chronic diseases than other groups [2-5]. While behavior change interventions have been effective for increasing physical activity in multiple population groups [6,7], participation can be difficult because of burdensome protocols, times constraints, and scheduling conflicts, particularly for those from marginalized backgrounds [8-11]. Mobile health (mHealth) approaches have helped mitigate some of these barriers by providing greater flexibility for participants, which can improve the reach of behavior change interventions [12]. mHealth can also improve the reach and scalability of interventions through automation, such as delivering content through automated messages and/or website- or app-based platforms. Mobile technologies are now highly prevalent among low-income groups, with more than 84% of adults in low-income households having a smartphone [13].

mHealth interventions have been effective for increasing physical activity in multiple population groups, but there has been less research in low-income groups [14-19]. The few prior mHealth studies addressing physical activity among low-income groups have shown promise for this approach [20-23]. However, there is a need for more mHealth research among these groups, especially studies that include both adults and youth. This family-inclusive approach could improve reach by allowing parents/caregivers and children to enroll together and may extend physical activity support within family networks. Another gap in prior work among low-income groups is that while interventions have used wearables and websites or apps and content is often tailored to the focus population, the extent to which the content is personalized or individualized for each participant has been limited in many studies [12,20-23]. Personalization better addresses the real-life circumstances and contexts people experience and can be accomplished in multiple ways, including customizing delivery timing, using participant information within content/messages, and using participant or environmental information to select and/or customize goals, feedback, prompts, or other content based on decision trees, algorithms, or even artificial intelligence [14,24-28]. Wearable physical activity trackers are particularly useful for personalizing content on physical activity goal monitoring, although there is a need for more research in low-income groups to investigate the integration of physical activity wearables with automated, personalized messaging using text messages [20-23].

This pilot study aimed to help address gaps in prior research by exploring a personalized, wearable-integrated, family-inclusive physical activity mHealth intervention among both adults and youth from low-income communities. The study used a beta-version intervention to collect initial information on the intervention approach to inform a more robust version of the intervention and evaluation in a future randomized clinical trial. The beta-version incorporated basic elements of personalization through integration of step-count and adaptive step-goal information into messages, use of branching logic to select messages based on step counts and goal achievement, 2-way question-and-response messaging, and use of participant names in messages. The evaluation focused on feasibility and acceptability (primary outcomes) while exploring preliminary evidence of physical activity behavior change.

## Methods

### Ethical Considerations

The study protocol was approved by Children’s Mercy Hospital’s Internal Review Board on August 24, 2021 (IRB study number STUDY00001939), and participants were compensated up to US $95 for completing study assessments. Prior to receiving the Garmin monitor, all participants provided written informed consent or child assent, and parents provided written parental permission. All data were deidentified prior to data storage and analyses. Study reporting was aligned with the Transparent Reporting of Evaluations with Nonrandomized Designs statement [29]. The only deviation from the original clinical trials registration was that step counts, rather than both step counts and physical activity minutes, were used as the physical activity outcome measure, as detailed below.

### Participants and Procedures

The study used a single-arm pre-post design, with all participants receiving the same active intervention, termed ActiveKC. Adults and youth were included, with those aged 8 years or older being eligible. Participants were required to be able to communicate in English and could be an adult, child, or parent–child dyad enrolled together. Either the participant or parent was required to have access to a smartphone. A priori eligibility criteria included that participants be insufficiently active, defined as not meeting step-count–based physical activity guidelines [30-32], because of the intervention’s focus on helping move individuals toward meeting these guidelines. Interested adults and youth wore a step tracker (Garmin Vívofit 4; Garmin International, Inc) for a 7-day run-in period and were excluded from the study if they averaged ≥7500 steps/day (adults) or ≥12,000 steps/day (youth). Individuals were also ineligible if they did not wear the Garmin device for ≥3 days during the run-in period because of perceived difficulty in their ability to receive the intervention content as intended, thus impacting internal validity. The selection of wear-time thresholds is detailed below.

Participants were recruited from 6 zip codes within eastern Kansas City, Missouri, that were considered low income by the study team because of having the highest scores on the Centers for Disease Control and Prevention Social Vulnerability Index [33] among all zip codes within Kansas City, Missouri. Social Vulnerability Index scores for the 6 selected zip codes ranged from 0.67 to 0.95 on a range of 0 (lowest vulnerability) to 1 (highest vulnerability) based on measures of socioeconomic status, household composition/disability, minority status and language, and housing and transportation. The 6 zip codes also had a large population of Black residents, with 44% to 88% of the residents being Black individuals. Recruitment occurred at COVID-19 vaccination and health screening community events hosted by neighborhood, youth, business, faith-based, and health organizations in collaboration with university health professional schools, and during well-child visits within a large hospital-based pediatric primary care health system serving primarily uninsured and underinsured families from the same communities where the events were held. At the community events, the study team operated a study-information booth and distributed study fliers to event attendees. In the health care system, study team members used a recruitment flier and script to offer enrollment to families while they were waiting to see their provider or just after seeing their provider. For both recruitment approaches, individuals were enrolled on site during the event or clinic visit, except in a few circumstances when a separate enrollment appointment was warranted so that a family member who was not present could be included. These separate appointments occurred in community locations, the study team’s office, or remotely over the telephone. Study enrollment occurred between September 2021 and February 2022.

### Intervention

The beta-version ActiveKC intervention was informed by prior qualitative research conducted with parents and children from the same low-income Kansas City communities of focus in this study [34]. A primary objective of the ActiveKC pilot project was to obtain participants’ input on design elements and intervention content while receiving a beta-version mHealth intervention. This participant input has been reported elsewhere.

**Figure 1:**
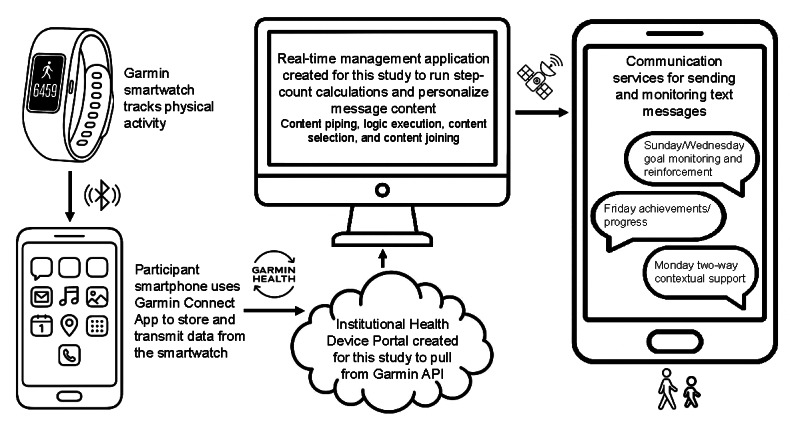
Overview of the ActiveKC Mobile Health System Architecture.

ActiveKC was a 7-week mHealth physical activity intervention that integrated the Garmin Vívofit 4 with automated intervention text messages developed by the study team. The messages were sent to the participant’s phone number using Microsoft Azure Communication Services and a real-time management application custom built for this project (Figure 1). For youth without a personal mobile phone, text messages were sent to the parent. Study staff helped set up the Garmin monitor, Garmin Connect smartphone app, and Garmin account; set the monitor to automatically synchronize with the Connect app; and demonstrated how to use the monitor and Connect app. Study staff monitored the data reports through the Garmin application programming interface (API) on a weekly basis and reached out to participants who did not have data within the prior 4 days. These participants were contacted via text message and phone call to provide technical assistance around synchronizing their Garmin watch with the Garmin Connect app on their smartphone (eg, because of problems with automatic synchronization). Participants could also request technical support from study staff.

The intervention focused on step counts because they are readily interpretable and preferred by many participants and because step counts and other measures of total physical activity have been consistently linked with health outcomes [35-40]. The intervention content used behavior change techniques [41] that were grounded in Social Cognitive Theory [42] and emphasized active living for building habitual physical activity into daily routines, including walking and movement throughout the day [43]. The automated text messages were sent 4 days/week and were personalized using the participant’s Garmin data obtained through the Garmin API, using 2-way messaging starting with a question that was used to tailor the content of the second message, and using the participant’s first name.

Step goals, monitoring, and reinforcement were core components of the intervention because of their grounding in Social Cognitive Theory [42] and because of consistent evidence showing their important role in behavior change [44]. The activity monitor tracked the participant’s daily steps and step goal, and the text messages focused on weekly goals, with each week spanning Sunday to Saturday. The daily step goal was set by the activity monitor based on the participant’s daily steps during their baseline week and adjusted each day using Garmin’s adaptive algorithm that increased or decreased the goal based on the participant’s step counts over the previous days. The weekly goal was the same for all participants: meeting their personalized daily goal on ≥4 days/week. Goal-monitoring messages were sent on Sundays (past-week review) and Wednesdays (midweek check-in). These messages were personalized based on step-count and goal-achievement data from the past week (past-week review; eg, “Congratulations Mallory, you met your step goal on 6 days this past week!”) or prior days in the current week (midweek check-in; eg, “Mallory, there’s still enough time to meet your step goal on 4 days this week. You just need 2 more days including today. You can do it!”). Conditional branching logic was used in these messages to include language that was reinforcing (eg, “You’re a natural!”) or encouraging (eg, “You can do it!”) based on whether the goal was met or on track to be met [41]. The Sunday messages also included a success story from a fictional participant in the intervention, targeting self-efficacy through vicarious learning with a similar model [45]. The Wednesday messages included a new tip on incorporating habitual physical activity into daily routines (eg, “Weekly activity tip: Sitting too much is bad for us even when we meet our step goal. Stand up every 20-30 minutes and move around for a minute or two!”). Friday messages were also personalized using data from the wearable tracker, revealing the number of miles the participant had walked since the start of the intervention and referencing an area or origin and destination with a similar distance (eg, the deepest part of the ocean). Throughout the study, participants were sent automated text messages to remind them to wear and/or synchronize the wearable tracker with the Garmin Connect app if data were not recorded on ≥3 consecutive days.

Each Monday, the participant received a 2-way message on a new topic related to multilevel contextual factors: knowledge, values, problem solving, planning, built environment, social support, and maintenance. The use of 2-way messaging allowed the system to send personalized information on the topic based on the participant’s response to the first message. The first message comprised a multiple-choice assessment of the context the participant was currently experiencing in relation to the topic. For example, the message for the problem-solving topic asked the participant to choose the top physical activity barrier they were facing that day or week, and the second message provided behavioral support for problem solving the selected barrier (eg, “When your energy is low and being active sounds like a chore, start small. Stand up, wiggle your limbs, and walk at a low pace until you are re-energized!”). Participants were then sent a link to a study web page with more information on the topic and on the unselected response options. Participants had 48 hours to respond to each Monday message to receive a response from the system.

### Measures

#### Sociodemographic Characteristics

Participant sociodemographic characteristics were collected via survey and included age, sex at birth, race/ethnicity, and adult or parent education (categorized as college degree or higher vs no college degree).

#### Feasibility

Enrollment and retention measures included the proportion of individuals who were enrolled out of all individuals who expressed interest and were assessed for eligibility, as well as the proportion of those enrolled who completed follow-up measurements (Garmin measures at the intervention midpoint and end point and survey measures at the intervention end point). Fidelity was based on the number of total messages per participant that were not received (eg, due to the participant’s phone service being disconnected), which was recorded through the messaging services provider. Adherence to wearing the Garmin device was based on the number of days the Garmin was worn across the intervention. The number of participants whom staff needed to contact to provide technical support was also tracked.

#### Acceptability

The primary items used to capture overall acceptability were: “How difficult or easy was it to use the program?” (“very difficult,” “somewhat difficult,” “somewhat easy,” or “very easy”); “How would you rate the frequency of the messages?” (“too few,” “just right,” or “too many”); “How much, if at all, did the program help you to be more active?” (“not at all,” “somewhat,” or “a lot”); and “Would you recommend that your family and friends use the program?” (“yes” or “no”). A total of 4 additional items had participants rate how helpful they perceived each of the primary 4 components of the intervention (step-tracker device, text-message content, ability to respond to messages, and web resources), with response options of “not at all,” “somewhat,” “very,” and “extremely.” Engagement with the text messages was captured as an additional indicator of acceptability based on text-message logs that tracked the number of times each participant responded to a 2-way message that asked for a reply, with up to 7 responses possible.

#### Physical Activity

Participants were asked to wear the Garmin Vívofit 4 step tracker throughout the intervention to facilitate intervention content and evaluation. Garmin step trackers have good validity for measuring step counts in adults and youth [46-48]. At the start of the screening period, study staff helped each participant set up a Garmin Connect account on their smartphone to facilitate automatic transfer of data into the Garmin API. Youth without a smartphone were connected to another device in the household (eg, tablet or parent’s smartphone). In the present analyses, daily step counts were evaluated over a 1-week baseline prior to the start of the intervention (week 0, also referred to as the run-in period), the intervention midpoint (weeks 3 and 4), and the end of the intervention (weeks 6 and 7). An exception was that the baseline period could have been 5 or 6 days if the participant was enrolled on a Monday or Tuesday. To account for nonwear time, 15-minute periods with a maximum motion intensity of 0, indicating no movement of the watch, were considered nonwear. Participant-level estimates for each assessment period were based on only valid days, defined as having ≥8 hours of wear time and ≥100 total steps. Participant-level estimates were set to missing if the participant did not have ≥3 valid days during the assessment period. These wear time criteria aimed to capture habitual physical activity while minimizing missing data [49,50]. The outcome variable of interest was an average daily step count for the participant at each assessment period. Minutes of physical activity was explored. However, those results are not reported because the Garmin model that was used calculated physical activity based on step counts and the results were redundant with the step-count outcome. Participants’ awareness of their physical activity levels was measured at baseline and at the end of the intervention with 1 item asking whether they generally knew how much physical activity or steps they obtained each day, with a 4-point response scale ranging from “strongly disagree” to “strongly agree.”

### Analysis

Descriptive statistics were summarized using frequencies, means, and SDs. Feasibility and acceptability benchmarks that were perceived to be reasonable targets in the current context were selected to help guide the interpretation of findings and were generally based on prior pilot behavioral and mHealth intervention studies [51,52]. These benchmarks included ≥60% for enrollment/eligibility, ≥80% for retention and fidelity, ≥70% for adherence, ≥80% for acceptability ratings, and ≥70% for engagement [51,52]. ANOVA and chi-square tests were used to investigate differences by eligibility/enrollment status, baseline physical activity, and level of engagement. Engagement as indicated by responses to messages was investigated as a categorical variable based on tertiles, which resulted in the following groups: replied to 0-1 messages, replied to 2-4 messages, and replied to 5-7 messages. We used 2-tailed *t* tests to investigate differences from preintervention to postintervention in participants’ awareness of the amount of physical activity they engaged in. Changes in daily step counts over time were explored using mixed-effects linear regression models, with participant entered as the nesting variable and time entered as a categorical fixed effect (coded as 0=baseline, 1=midpoint, and 2=end point) to test differences at both the midpoint and end point. Models were adjusted for daily wear time (minutes/day), number of valid wear days, and proportion of valid wear days that were weekdays during each measurement period as time-varying fixed effects. These models were explored in the full adult and youth sample as well as post hoc by subgroup based on meeting less than two-thirds of the recommended daily steps at baseline based on the physical activity guidelines (<5000 steps/day for adults and <8000 for youth) [30-32], which is aligned with clinical trials that have focused on individuals with lower activity. These participants were of interest given the low levels of physical activity in the US population, particularly among low-income groups [5]. There is also greater potential for improvement among these individuals and a need for interventions that can help individuals move from low to moderate levels of physical activity [1,4,5]. The same models were also explored within subgroups based on level of engagement with the messages asking for a response, to capture dose-response and help inform whether observed increases in step counts were potentially attributable to greater exposure to intervention content. An intent-to-treat approach was used by including those lost to follow-up in the mixed-effects models and using restricted maximum likelihood estimation to account for the missing Garmin data. Effect sizes (Cohen *d*) were calculated by dividing the mean change in daily steps from baseline to the end of the intervention by the SD in this change. Given this pilot study was not powered to detect effectiveness, interpretations of the data were focused more on effect sizes than *P* values. All analyses were conducted separately for adults and youth using SPSS (version 27.0, SPSS Inc).

## Results

### Feasibility

**Figure 2:**
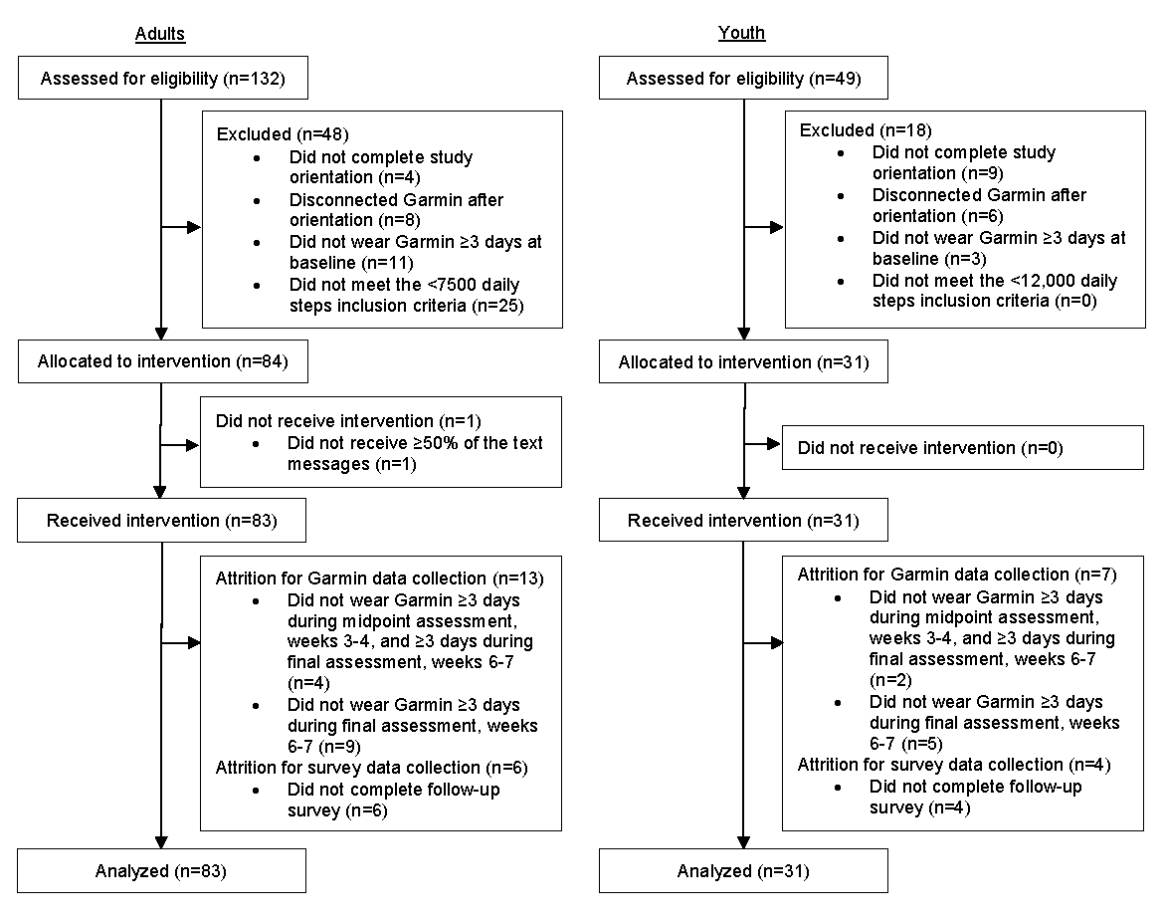
Study CONSORT (Consolidated Standards of Reporting Trials) diagram.

Figure 2 presents study enrollment, exclusions, and retention information based on the CONSORT (Consolidated Standards of Reporting Trials) guidelines. Among adults, 64% (84/132) of those assessed for eligibility were enrolled into the intervention, although 1 participant did not receive most of the intervention text messages because of their phone being disconnected or because they changed phone numbers. Similar enrollment rates were observed for youth, with 63% (31/49) of those assessed for eligibility being enrolled. A total of 19% (25/132) of adults were ineligible because of the step-count criteria during the run-in period, whereas no youth were ineligible based on these criteria. No individuals who were screened for eligibility were ineligible because of lack of access to a smartphone.

The analytic sample comprised 83 adults and 31 youth. Among adult participants, 88% (73/83) were female, 84% (70/83) were Black or African American individuals, and the mean age was 50 years (range 19-88 years) (Table 1). A total of 77% (24/31) of the youth participants were female, 77% (24/31) were Black or African American individuals, and the mean age was 13 years (range 8-17 years). A total of 84% (26/31) of the youth participants had a parent who also participated. The eligible/enrolled (n=83) and ineligible/not enrolled (n=48) adults were similar with regards to age and race/ethnicity, although enrolled adults were significantly (*P*<.05) more likely to have a college degree (30/80, 37% vs 7/46, 15%) and nonsignificantly more likely to be female (73/83, 88% vs 39/48, 75%; Table S1 in Multimedia Appendix 1). The eligible/enrolled (n=31) and ineligible/not enrolled (n=18) youth were similar with regards to age, although enrolled youth were significantly more likely to be female (24/31, 77% vs 4/12, 33%), significantly more likely to be Black individuals (24/31, 77% vs 9/17, 53%), and nonsignificantly more likely to have a parent with a college degree (6/29, 21% vs 0/12, 0%; Table S2 in Multimedia Appendix 1). For 48% (15/31) of the youth participants, messages were sent to the parent’s phone. These youth were significantly younger than those whose messages were sent to their own phone (mean age 10.5, SD 1.9 years vs mean age 15.1, SD 1.9 years) and nonsignificantly more likely to have a parent with a college degree (4/14, 29% vs 1/14, 7%), although the 2 groups were similar with regards to sex, race/ethnicity, or parental education (Table S2 in Multimedia Appendix 1).

Retention rates were 95% (79/83) among adults and 94% (29/31) among youth for the step-tracker midpoint assessment, and 84% (70/83) among adults and 77% (24/31) among youth for the step-tracker end point assessment. Retention was 93% (77/83) among adults and 87% (27/31) among youth for the survey end point assessment. Across the 3955 messages that were sent, a total of 1.1% (45/3955) failed to be delivered, largely because of mobile phone numbers being inactive for a period of time. No participants experienced adverse events related to the intervention.

For adherence to wearing the step tracker, adults and youth had a mean of 46 (SD 13) valid wear days and 44 (SD 13) valid wear days, respectively, over the 54- to 56-day period spanning the 5- to 7-day baseline and 7-week intervention (ie, 45/56, 82% and 44/56, 79%). Wear time at each study assessment period is summarized in Table S3 in Multimedia Appendix 1. Study staff contacted 10 total participants to troubleshoot technical problems, all of which were related to a lack of recent Garmin data. Of these, 9 participants were documented as experiencing technical problems related to their Garmin monitor synchronizing with the Garmin app on their smartphone, while the other participant was believed to have disengaged from wearing the study monitor.

**Table 1:** Participant sociodemographic characteristics.

Characteristic		Adults (n=83)		Youth (n=31)
Age (years), range		19-88		8-17
Age (years), mean (SD)		49.6 (17.0)		12.9 (3.0)
Female, n (%)		73 (88)		24 (77)
**Race/ethnicity, n (%)**				
	Black/African American	70 (84)	24 (77)	
	White non-Hispanic	11 (13)	6 (19)	
	Hispanic/Latino	0 (0)	0 (0)	
	Other	2 (2)	1 (3)	
No college degree, n (%)^a^		50 (63)		23 (79)^b^

^a^Percentages were calculated based on nonmissing responses (3 and 2 participants had missing responses in the adult and youth columns, respectively).

^b^Refers to parent education.

### Acceptability

Among adults, 88% (69/78) indicated the intervention was somewhat or very easy to use, 83% (65/78) rated the frequency of messages as just right, 99% (77/78) believed the program helped them be more active, and 97% (75/77) indicated they would recommend the program to family and friends (Table S3 in Multimedia Appendix 1). Among youth, 96% (27/28) indicated the program was somewhat or very easy to use, 93% (26/28) rated the frequency of messages as just right, 100% (28/28) believed the program helped them be somewhat or a lot more active, and 100% (28/28) indicated they would recommend the program to family and friends. The step tracker device was the highest rated component of the intervention, followed by the text message content and ability to respond to messages, and these results were similar for adults and youth (Table S3 in Multimedia Appendix 1).

Adults replied to a mean of 2.6 (SD 2.2) of the 7 text messages that asked for a reply within the allotted 48-hour response period. Youth replied to a mean of 3.2 (SD 2.7) messages. Among adults, those who had a child who also participated replied to more messages than those without a participating child (mean 3.3, SD 2.2 messages vs mean 2.4, SD 2.2 messages), although this difference was nonsignificant (2-tailed *t*_81_=1.5; *P*=.12). Adults who replied to more messages were significantly (*P*<.05) more likely to be White non-Hispanic and have a college degree, and nonsignificantly more likely to be female and younger in age (Table S1 in Multimedia Appendix 1). Youth who replied to more messages, including those with messages sent to the parent’s phone, were similar to those who replied to fewer messages with regard to age, sex, race/ethnicity, and parent education (Table S2 in Multimedia Appendix 1).

### Physical Activity

Adults and youth had a mean of 4761 (SD 1719) daily step counts and 6618 (SD 2229) daily step counts at baseline, respectively. Adults and youth experienced small and nonsignificant increases in their daily step counts from baseline to the end of the intervention (weeks 6-7) of a mean of 240 (SD 1799; *d*=0.13) steps/day and 413 (SD 2698; *d*=0.15) steps/day, respectively, based on the regression analysis (Table 2).

Exploratory analyses suggested that participants with lower daily step counts at baseline experienced larger increases over the course of the intervention, with adults and youth who entered the study with a daily step count that was less than two-thirds of the recommended daily steps experiencing nonsignificant increases of a mean of 609 (SD 1534; *d*=0.40) steps/day and 1406 (SD 2445; d=0.58) steps/day by the end of the intervention, respectively (Table 2). This reflected increases of 18% and 26% over baseline. As shown in Tables S1 and S2 in Multimedia Appendix 1, both adults and youth with less than two-thirds of the recommended daily steps at baseline were similar to those with at least two-thirds of the recommended daily steps at baseline with regard to age, sex, race/ethnicity, and adult education.

Exploratory analyses also suggested that participants who had higher engagement with the intervention messages experienced larger increases over the course of the intervention, with adults and youth in the highest tertile of engagement experiencing nonsignificant increases of a mean of 584 (SD 1744; *d*=0.33) steps/day and 941 (SD 2465; *d*=0.38) steps/day by the end of the intervention, respectively (Table 2). This reflected increases of 12% (584/4838) and 15% (941/6321) over baseline. Among youth, data suggested that larger increases in daily steps were experienced by those whose parent was receiving messages about the child (mean 530, SD 2690 steps/day; *d*=0.20), rather than the child receiving the messages on their own mobile phone.

Increases were observed in participants’ awareness of their physical activity levels, from a mean of 2.7 (SD 1.0) to 3.1 (SD 0.8) in adults (2-tailed *t*_53_=–2.6; *P*=.01) and a mean of 2.6 (SD 0.7) to 2.9 (SD 0.7) in youth (2-tailed *t*_16_=–1.8; *P*=.10).

**Table 2:** Daily step counts over the course of the intervention. Estimated means, SEs, unstandardized regression coefficients (β), 95% CIs, and *P* values were estimated from mixed-effects linear regression models with adjustment for daily wear time, number of wear days, and proportion of wear days that were weekdays during each measurement period. Each subsample was investigated in a separate model.

Sample and subsample		Frequency, n	Daily step count, estimated mean (SE)				Change in daily steps at intervention midpoint			Change in daily steps at intervention end point	
			Baseline^a^	Intervention midpoint^b^	Intervention end point^c^	β (95% CI)		*P* value	β (95% CI)		*P* value
**Adults**											
	Full sample	83	4761 (198)	4908 (216)	5000 (244)	148 (–448 to 743)		.62	240 (–387 to 866)		.45
**By baseline activity**											
	≥5000 daily steps at baseline	36	6379 (128)	6256 (299)	6124 (329)	–123 (–793 to 547)		.71	–256 (–971 to 460)		.48
	<5000 daily steps at baseline	47	3404 (162)	4049 (210)	4013 (272)	645 (105 to 1185)		.02	609 (–30 to 1247)		.06
**By engagement**											
	Replied to 0-1 messages	32	4830 (337)	4925 (316)	4822 (357)	94 (–843 to 1031)		.84	–8 (997 to 981)		.99
	Replied to 2-4 messages	32	4821 (329)	4909 (344)	4797 (376)	89 (–913 to 1091)		.86	–24 (–1057 to 1010)		.96
	Replied to 5-7 messages	19	4838 (353)	4740 (491)	5423 (564)	–99 (–1377 to 1180)		.88	584 (–784 to 1952)		.39
**Youth**											
	Full sample	31	6618 (411)	6517 (366)	7031 (482)	–101 (–1221 to 1018)		.86	413 (–877 to 1703)		.52
**By baseline activity**											
	≥8000 daily steps at baseline	9	9590 (317)	7150 (357)	7765 (323)	–2440 (–3482 to –1398)		<.001	–1825 (–2805 to –846)		.002
	<8000 daily steps at baseline	22	5386 (289)	6194 (502)	6793 (654)	807 (–389 to 2004)		.18	1406 (–94 to 2907)		.06
**By engagement**											
	Replied to 0-1 messages	11	6913 (547)	7005 (911)	6125 (1212)	91 (–2236 to 2419)		.93	–788 (–3959 to 2382)		.57
	Replied to 2-4 messages	9	6980 (681)	5974 (820)	7057 (1042)	–1006 (–3490 to 1478)		.40	77 (–2732 to 2885)		.95
	Replied to 5-7 messages	11	6321 (596)	6531 (409)	7262 (577)	209 (–1333 to 1752)		.78	941 (–827 to 2709)		.28
**By phone used**											
	Child’s phone	16	7485 (759)	6254 (688)	6809 (978)	–1230 (–3701 to 1240)		.31	–676 (–3518 to 2165)		.63
	Parent’s phone	15	6397 (594)	6425 (462)	6927 (549)	28 (–1512 to 1568)		.97	530 (–1133 to 2192)		.52

^a^Baseline=week 0

^b^Intervention midpoint=weeks 3-4.

^c^Intervention end point=weeks 6-7.

## Discussion

### Overview

Findings generally supported high feasibility and acceptability of the beta-version mHealth intervention among adult and youth participants from marginalized, low-income communities. Most benchmarks were achieved for enrollment/eligibility, retention, fidelity, adherence, and acceptability, although level of engagement based on replies to messages was moderate overall and highly variable. The step-count data provided preliminary evidence in support of increased physical activity, particularly among those who had a low amount of physical activity at baseline (less than two-thirds of the daily recommendation) or who were more engaged in the intervention. The engagement and physical activity findings were most promising among youth, particularly those who were younger and participated as part of a dyad whereby messages were sent to the parent. These findings warrant expansion of the beta-version intervention to further incorporate end-user preferences around content and personalized components along with rigorous evaluation using a randomized controlled trial design.

### Feasibility

Feasibility was supported across all indicators for both adults and youth, including rates of enrollment/eligibility (31/49, 63% to 84/132, 64%), retention (24/31, 77% to 77/83, 93%), fidelity of message delivery (3910/3955, 99%), and adherence to wearing the step tracker (44/56, 79% to 46/56, 82% of days). All individuals screened had a smartphone, which reflects the growing ubiquity in smartphone use among low-income populations [13] and supports the potential for mHealth interventions to have high reach among this population group. The exclusion of 19% (25/132) of adults because of already meeting the physical activity guideline aligns with the low levels of physical activity in the US population and generally lower levels among low-income groups [4], although it was alarming that none of the youth who were screened were already meeting the physical activity guideline, highlighting the particular importance of efforts to increase physical activity among youth from these communities. The enrolled participants generally reflected the target population based on sociodemographic factors, although findings suggest a need to improve reach among boys, men, and adults with lower education. Engaging men in health behavior interventions is a known challenge and may warrant interventions tailored for adult men from specific population groups [53]. The low rate of enrollment among boys warrants further investigation and differs from other family-based interventions and interventions among children, which have generally had similar reach among boys and girls [54,55]. The retention rate in youth for the final physical activity assessment period warrants improvement, with potential strategies including incentivization tied to wearing the monitor for specific time periods and even using a separate monitor (eg, research-grade accelerometer) for baseline and end point evaluation measurement given the need to retain participants in assessments even if they disengage from the intervention.

The high level of adherence to wearing the step tracker among adults and youth can also be viewed as a marker of engagement in the intervention, supporting both feasibility and acceptability. Prior studies have observed similar levels of adherence during relatively brief (eg, 12-week) interventions, with poorer adherence over longer periods of time and at follow-up periods [56], indicative of the challenges with ongoing adherence to wearing step trackers. Adherence is especially important in wearable-integrated interventions that rely on activity data. Given the vast body of evidence supporting self-monitoring as one of the most important components in physical activity and behavior change interventions [44,57], there is a need for more research on longer-term adherence to consumer wearable trackers [58]. Future mHealth research should consider testing whether intermittent periods of wearing the tracker can provide benefits that translate to periods of nonwear and testing “back-up” strategies for supporting goal setting and monitoring during periods of nonwear.

Many participants experienced technical problems with the step tracker, primarily with the tracker failing to regularly synchronize with the Garmin app on their smartphone. This challenge has been documented across many prior studies and monitor brands and models and thus is a critical issue facing wearable-integrated mHealth research [59]. Study staff were able to work with many of these participants to achieve resolution, although anecdotal evidence that we were unable to quantify suggested that some problems were recurring and that some participants needed to regularly engage with their Garmin app to ensure proper synchronizing. The exact source of the problem could vary and was often unknown, and the process for implementing and testing solutions could vary somewhat across smartphone software type and version, requiring staff to develop detailed guidance protocols to capture the different scenarios encountered. Overall, there is a need for more research to guide effective and efficient technical support for wearable-based physical activity interventions to reduce missing data and prevent disruptions in delivery of tailored intervention content, particularly among population groups with less experience with technology.

### Acceptability

The high overall acceptability ratings of the intervention among adults and youth (65/78, 83% to 28/28, 100%) show that participants generally enjoyed the automated intervention. The step tracker device was a favorite aspect of the intervention, especially among youth. This was likely attributable to the behavioral monitoring features it provided, given participants became more aware of their physical activity during the intervention and given the entry-level device had limited additional features (eg, no internet connectivity or text message alerts). The acceptability ratings suggested that participants liked the message frequency of approximately once per day on 4 days of the week, which is similar to the messaging frequency used in some prior physical activity interventions [15,20,60]. This somewhat frequent delivery of messages helps facilitate timely interaction and check-ins, allowing content to be personalized to experiences related to that day or even time of day. However, the acceptability ratings of the text messages were lower than the overall acceptability ratings for the intervention, and engagement based on replies to messages was moderate and highly variable across participants. These findings indicate a need for refined message content and potentially message types and targets. Qualitative research from this study sample, which is being reported elsewhere in more detail, suggested that participants generally preferred personalized messages over other messages, particularly the 2-way messages and the messages that were customized using their Garmin data, although present findings suggest that even these message types warrant further optimization. Future studies should use additional user-centered design strategies to collect participant input on specific messages and personalization components to optimize content, such as iterative prototyping and co-design activities [61-64]. There is also a need to explore additional approaches for personalization within this study population, such as integrating periodic questionnaires, ecological momentary assessments, location tracking, and/or meteorological monitoring [26-28]. Study designs such as microrandomized and multifactorial trials allow for testing different types of messages and personalization elements and thus are ideal next steps for optimizing this and similar interventions [65].

Participant engagement with intervention content is a common concern in behavioral interventions, including those using mHealth [66]. The participant characteristics among those who were more likely to engage in responding to messages can help to inform the population groups that future versions of the intervention should focus on, although research is also needed to identify strategies for improving engagement among groups that may be more difficult to engage. The present engagement findings generally suggest that wearable-integrated, text message–based mHealth interventions may be most promising among youth and among younger adults, and that additional strategies are needed to support engagement among older adults, Black individuals, individuals with lower education, and men. Features that have been shown to increase engagement include personalization, which was a focus of this study but through basic initial elements, as well as gamification, social interaction, and live human integration (eg, coaching) [67,68]. Present findings also suggest that participating together as a parent–child dyad may result in higher engagement, which warrants more family-based research in this area. Theory-based frameworks such as Family Systems Theory [69] can guide family-based physical activity interventions and be used to inform personalized content, so their use should be investigated within this context to better address engagement from both the parent and child. It is also important to consider that, given the flexibility and scalability of mHealth tools, lower levels of engagement may be acceptable, especially if behavioral outcomes are still achieved.

The web-based content was rated as the least helpful aspect of the intervention, warranting more exploration of strategies for providing helpful behavior change content beyond what is provided in the text messages themselves. Through qualitative research in this study sample, we discovered that some participants were concerned about clicking on website links that were provided in the text messages, which may have impacted acceptability. Future studies should track website metrics such as visits by each participant, which we have had success doing in other studies through use of participant-specific URLs for each study web page. More research is needed to test strategies for increasing engagement in asynchronous intervention content, which could include personalization of content within web pages, enhancements to credibility, enhancement to aesthetics, multimedia content (eg, pictures, videos, and audio), gamification, peer-to-peer engagement, and/or reinforcing engagement [70,71].

### Physical Activity

The magnitude of the changes in daily step counts was small and not likely to be meaningful in the full sample, although the changes appeared meaningful for participants who were more engaged in the intervention or who were further from meeting the recommended daily steps at baseline. The association between engagement and physical activity has also been observed in prior research [72] and suggests that the observed changes were more likely to be attributable to intervention participation than to an external or confounding factor. These exploratory findings were also useful in informing who may be more likely to benefit from future iterations of the intervention, pointing to those with low levels of activity (meeting less than two-thirds of the guideline) and parent–child dyads (ie, children with parent involvement). The approximately 500 steps/day to approximately 1400 steps/day changes in daily steps among these subgroups of youth are comparable to prior digital health interventions in youth from different population groups and to amounts recommended for health improvements, which reflect changes of approximately 1000 steps/day (reflecting approximately 10 minutes/day of moderate-to-vigorous physical activity [MVPA]) [24,30,73,74]. Similarly, the approximately 500 steps/day to approximately 600 steps/day changes in adults are comparable to prior studies in adults and are within the range of 500 steps/day to 600 steps/day (reflecting approximately 30-35 minutes/week of MVPA) that has been shown to produce clinically meaningful health improvements [1,15,16,31,32,75]. However, caution should be used when considering these changes alongside findings from prior studies given the pilot nature of this study and lack of a control group. Overall, the physical activity findings warrant additional research on mHealth physical activity interventions among low-income populations to focus on optimization and effectiveness testing in randomized controlled trials.

### Strengths and Limitations

Study strengths and innovations included the focus on a population group that has been historically underrepresented in research and the integration of device-based physical activity data with automated messages that were personalized based on goal achievements. The inclusion of both adults and youth was a study strength given this approach can increase reach and allow families to participate together. However, the intervention content did not differ between adults and youth in this beta-version intervention, and qualitative data collected as part of this study (reported separately) suggest future iterations of the intervention should heavily tailor the content for different age groups (eg, children, adolescents, parents, and other adults). While the intervention content was informed through community engagement, more work is needed to refine the content through additional cocreation strategies to optimize its relevance for the population group. The study design did not support investigation of which intervention components or text messages had the greatest impact on engagement and physical activity. Since message frequency did not vary across weeks or across participants, we were not able to explore the impact of message frequency. We were unable to track whether participants viewed text messages or web pages, which are important aspects of engagement that should be assessed in future research. Survey measures generally relied on single-item ratings that did not have established psychometric properties and thus should be interpreted cautiously. The lack of a control group prevented the ability to distinguish the effects of the intervention from natural changes in physical activity that may have occurred over time and thus limited the ability to make causal inferences. Few of the daily step increases observed were statistically significant, which was likely due to the small sample size and wide CIs, so results should be confirmed in larger samples. The step count data collected from the Garmin device did not provide insights into activity intensity, so we were unable to distinguish light activity from MVPA. Since the study focused on a low-income group, the results may not generalize to other population groups.

### Conclusions

This study showed high feasibility and acceptability for using basic personalized mHealth physical activity intervention components that integrate wearable step trackers with automated text messaging among adults and youth from marginalized, low-income communities. More robust development and testing of this type of intervention appears particularly promising for children with low physical activity through involvement from a parent (ie, dyadic approach). The daily step increases associated with the brief beta-version intervention warrant larger-scale effectiveness research in randomized controlled trials. Given the importance of content personalization, future research is warranted to evaluate additional strategies for delivering highly personalized and engaging content for marginalized population groups.

## Appendix

Multimedia Appendix 1 Comparison of participant sociodemographic characteristics, adults.

## Abbreviations

API: application programming interface
CONSORT: Consolidated Standards of Reporting Trials
mHealth: mobile health
MVPA: moderate-to-vigorous physical activity

## Data Availability

The datasets generated or analyzed during this study are available from the corresponding author on reasonable request.
