# Supplementary material for: Automated Physical Activity Support for Adults and Youth From Low-Income Communities: Single-Arm Pilot Study
Source: JMIR Mhealth Uhealth. 2026 Jun 5;14:e76991. doi: 10.2196/76991 (PMC13282599; doi:10.2196/76991)
Supplement: Multimedia Appendix 1 [file mhealth_v14i1e76991_app1.docx]

| *Table S1. Comparison of participant sociodemographic characteristics, adults* | | | | | | | |
| --- | --- | --- | --- | --- | --- | --- | --- |
|  | By enrollment | | By baseline activity | | By engagement | | |
|  | Screened ineligible | Eligible/  Enrolled^a^ | ≥5000 daily steps at baseline | <5000 daily steps at baseline | Replied to 0-1 messages | Replied to 2-4 messages | Replied to 5-7 messages |
| N with available data | 46-48 | 80-83 | 34-36 | 46-47 | 30-32 | 31-32 | 19 |
| Mean (SD) age, years | 49.3 (13.8) | 49.6 (17.0) | 46.7 (14.3) | 51.7 (18.6) | 53.5 (18.4) | 49.8 (15.0) | 42.5 (16.1) |
| Female, number (%) | 36 (75%) | 73 (88%) | 30 (83%) | 43 (92%) | 26 (81%) | 29 (91%) | 18 (95%) |
| Race/Ethnicity, number (%) |  |  |  |  |  |  |  |
| Black/African American | 42 (82%) | 70 (84%) | 30 (83%) | 40 (85%) | 32 (100%)^b^ | 27 (84%) | 11 (58%) |
| White non-Hispanic | 3 (6%) | 11 (13%) | 5 (14%) | 6 (13%) | 0 (0%) | 4 (13%) | 7 (37%) |
| Hispanic/Latino | 3 (6%) | 0 (0%) | 0 (0%) | 0 (0%) | 0 (0%) | 0 (0%) | 0 (0%) |
| Other | 3 (6%) | 2 (2%) | 1 (3%) | 1 (2%) | 0 (0%) | (3%) | 1 (5%) |
| No college degree, number (%) | 39 (85%)^b^ | 50 (63%) | 21 (62%) | 29 (63%) | 24 (80%)^b^ | 17 (55%) | 9 (47%) |
| ^a^84 participants were enrolled but 1 did not receive the intervention; ^b^Indicates significant differences (P<.05) across groups based on omnibus test from chi squared test or ANOVA. | | | | | | | |

| *Table S2. Comparison of participant sociodemographic characteristics, youth* | | | | | | | | | |
| --- | --- | --- | --- | --- | --- | --- | --- | --- | --- |
|  | By enrollment | | By baseline activity | | By engagement | | | By phone used | |
|  | Screened ineligible | Eligible/  enrolled | ≥8000 daily steps at baseline | <8000 daily steps at baseline | Replied to 0-1 messages | Replied to 2-4 messages | Replied to 5-7 messages | Child’s phone | Parent’s phone |
| N with available data | 12-17 | 28-31 | 9 | 19-22 | 11 | 8-9 | 9-11 | 14-16 | 14-15 |
| Mean (SD) age, years | 12.0 (2.5) | 12.9 (3.0) | 11.3 (3.0) | 13.5 (2.8) | 13.2 (2.7) | 11.1 (2.8) | 14.0 (2.9) | 15.1 (1.9)^b^ | 10.5 (1.9) |
| Female, number (%) | 4 (33%)^b^ | 24 (77%) | 7 (78%) | 17 (77%) | 8 (73%) | 8 (89%) | 8 (73%) | 12 (75%) | 12 (80%) |
| Race/Ethnicity, number (%) |  |  |  |  |  |  |  |  |  |
| Black/African American | 9 (53%)^b^ | 24 (77%) | 7 (78%) | 17 (77%) | 9 (82%) | 6 (67%) | 9 (82%) | 12 (75%) | 12 (80%) |
| White non-Hispanic | 0 (0%) | 6 (19%) | 2 (22%) | 4 (18%) | 2 (18%) | 2 (22%) | 2 (18%) | 4 (25%) | 2 (13%) |
| Hispanic/Latino | 2 (12%) | 0 (0%) | 0 (0%) | 0 (0%) | 0 (0%) | 0 (0%) | 0 (0%) | 0 (0%) | 0 (0%) |
| Other | 6 (35%) | 1 (3%) | 0 (0%) | 1 (5%) | 0 (0%) | 1 (11%) | 0 (0%) | 0 (0%) | 1 (7%) |
| No college degree, number (%)^a^ | 12 (100%) | 23 (79%) | 8 (89%) | 15 (79%) | 9 (82%) | 7 (88%) | 7 (78%) | 13 (93%) | 10 (71%) |
| ^a^Refers to parent education; ^b^Indicates significant differences (P<.05) across groups based on omnibus test from chi squared test or ANOVA. | | | | | | | | | |

| *Table S3. Monitor adherence and post intervention ratings of intervention components* | | | | | | | | |
| --- | --- | --- | --- | --- | --- | --- | --- | --- |
|  |  | Adults, Mean (SD) | | |  | Youth, Mean (SD) | | |
| Adherence measures | N | Baseline | Intervention midpoint | Intervention end point | N | Baseline | Intervention midpoint | Intervention end point |
| Number of valid days per week | 83 | 5.5 (1.3) | 6.1 (1.8) | 5.1 (2.6) | 31 | 5.2 (1.3) | 5.6 (2.1) | 4.7 (2.8) |
| Number of valid hours per day^a^ | 83 | 20.9 (3.0) | 21.4 (2.8) | 21.0 (3.2) | 31 | 21.6 (2.7) | 21.9 (2.2) | 22.0 (2.0) |
|  |  | Adults, N (%) of respondents | | |  | Youth, N (%) of respondents | | |
| Acceptability measures | N | Not helpful | Somewhat helpful | Very/extremely helpful | N | Not helpful | Somewhat helpful | Very/extremely helpful |
| Wearable step tracker | 77 | 0 (0%) | 13 (17%) | 64 (83%) | 25 | 0 (0%) | 1 (4%) | 24 (96%) |
| Text message content | 78 | 2 (3%) | 20 (26%) | 56 (72%) | 28 | 0 (0%) | 8 (29%) | 20 (71%) |
| Ability to respond to messages | 78 | 4 (5%) | 23 (30%) | 51 (65%) | 28 | 1 (4%) | 7 (26%) | 19 (70%) |
| Web resources | 77 | 7 (9%) | 28 (36%) | 42 (55%) | 27 | 2 (7%) | 16 (59%) | 9 (33%) |
|  | N | Very/somewhat difficult | Somewhat easy | Very easy | N | Very/somewhat difficult | Somewhat easy | Very easy |
| Ease of using the program | 78 | 9 (12%) | 32 (41%) | 37 (47%) | 28 | 1 (4%) | 5 (18%) | 22 (79%) |
|  | N | Too many | Too few | Just right | N | Too many | Too few | Just right |
| Frequency of messages | 78 | 7 (9%) | 6 (8%) | 65 (83%) | 28 | 1 (4%) | 1 (4%) | 26 (93%) |
|  | N | Not at all | Somewhat | A lot | N | Not at all | Somewhat | A lot |
| Program helped increase physical activity | 78 | 1 (1%) | 35 (45%) | 42 (54%) | 28 | 0 (0%) | 11 (39%) | 17 (61%) |
| ^a^On valid days, can include sleep time.  Baseline=Week 0; Intervention midpoint=Weeks 3-4; Intervention end=Weeks 6-7. | | | | | | | | |
